# Efficacy and Safety of the Sandwich Method in Patients with Benign Prostate Hyperplasia: Bipolar Transurethral Resection with GreenLight Vaporization

**DOI:** 10.3390/jcm11051276

**Published:** 2022-02-26

**Authors:** Tsu-Chen Lin, Po-Chih Chang, I-Hung Shao, Yu Chen, Hsin-Chieh Huang, Yu-Chao Hsu, Ming-Li Hsieh

**Affiliations:** 1 Division of Urology, Department of Surgery, Linkou Chang Gung Memorial Hospital, Taoyuan City 333, Taiwan; jamie8815@gmail.com (T.-C.L.); henrychang@cgmh.org.tw (P.-C.C.); ehomeshao68@gmail.com (I.-H.S.); yu.iok2681@gmail.com (Y.C.); hhc3191@cgmh.org.tw (H.-C.H.); yuchaohsu@gmail.com (Y.-C.H.); 2 College of Medicine, Chang Gung University, Taoyuan City 333, Taiwan

**Keywords:** benign prostate hyperplasia, bipolar-transurethral resection of the prostate, GreenLight photoselective vaporization of the prostate, large prostate, transurethral surgery of prostate

## Abstract

Benign prostate hyperplasia (BPH) refers to the nonmalignant enlargement of the transition zone of the prostate gland. While holmium laser enucleation of the prostate and open simple prostatectomy are effective in the management of patients with large prostates, they have some limitations. Thus, this study aimed to analyze the efficacy and safety of the sandwich method of bipolar transurethral resection of the prostate (B-TURP) and GreenLight photoselective vaporization of the prostate (GLPVP) in patients with large prostates. Patients diagnosed with BPH who underwent the sandwich method with B-TURP and GLPVP from 2015 to 2020 were included. Efficacy analyses included the change in the uroflowmetry results in both group A (prostate volume < 80 g) and group B (prostate volume ≥ 80 g), and complication analyses included perioperative complications, early postoperative complications at three months and late postoperative complications at 12 months. The cohort comprised 188 and 44 patients in groups A and B, respectively. The prostate volume of groups A and B were 50.83 ± 14.14 g and 102.03 ± 19.36 g (*p* < 0.001), respectively. The peak (Q_max_) and average (Q_avg_) flow rates were comparable between the two groups. The only significant difference noted was in the postoperative post-void residual (PVR) urine. Improvement was seen in all the variables including the Q_max_, Q_avg_ and PVR urine in each group. No patient experienced perioperative complications. Analysis of the overall one-year complication rate showed no significant difference between the two groups. The sandwich method of B-TURP and GLPVP may be feasible for the management of patients with large prostate.

## 1. Introduction

Benign prostate hyperplasia (BPH) refers to the nonmalignant enlargement of the transition zone of the prostate gland, mainly causing an increase in the size of the stromal tissue [1]. It is one of the main causes of lower urinary tract symptoms (LUTS). This disease is associated with multiple risk factors, including non-modifiable factors, genetics, aging and geography [2,3]. Thus, its prevalence increases with age. It affects more than 20% of 30 to 79 year-old men in the United States, and over 80% of 70 year-old men [4]. According to a systematic review and meta-analysis, the combined lifetime prevalence is estimated to be 26.2% [5]. Its high prevalence is also associated with increased healthcare costs and other medical morbidities [5].

According to the American Urologic Association (AUA) guidelines in 2021, surgical management is recommended in patients with refractory urinary retention, renal insufficiency, recurrent gross hematuria or bladder stones due to BPH, recurrent urinary tract infections or LUTS refractory to other therapies, including medical therapy [6]. Various surgical treatments have been developed for BPH management, including transurethral surgery, minimally invasive surgeries, open simple prostatectomy (OSP) and robotic assisted surgeries [6,7]. Bipolar transurethral resection of the prostate (B-TURP) and photoselective vaporization of the prostate (PVP) are two of the possible effective surgical treatments. Both are recommended for use in patients with a prostate size of 30 to 80 mL according to the European Association of Urology (EAU) guidelines [8].

Currently, high-quality studies on the management of patients with large prostates are limited [8]. Two of the methods that have been shown to be effective in this population are holmium laser enucleation of the prostate (HoLEP) and OSP; however, both of these methods have limitations [9]. Thus, this study aimed to analyze the efficacy and safety of the sandwich method using B-TURP and GreenLight PVP (GLPVP) in patients with large prostates.

## 2. Materials and Methods

### 2.1. Study Population

A retrospective analysis was conducted at Linkou Chang Gung Memorial Hospital (CGMH), a tertiary medical center in Taiwan. Patients diagnosed with BPH who underwent the sandwich method with B-TURP and GLPVP from 2015 to 2020 were included in the analysis. Patients with missing information or history of prostate cancer were excluded from the study. This study was approved by the ethics committee of Linkou CGMH (IRB number: 202101983B0).

### 2.2. Surgical Operation

All patients underwent the sandwich method with B-TURP and GLPVP. GLPVP was performed using the 180-W GreenLight XPS laser system. GLPVP was first performed for prostatic adenoma tissue ablation through vaporization to its maximal depth, until limited efficacy and necrotic tissue was observed. B-TURP was then utilized to further resect the prostatic and necrotic tissues, thereby exposing further prostatic adenoma, while collecting prostate specimens for pathologic examination. The resultant adenoma tissue was ablated, and hemostasis was achieved using GLPVP.

### 2.3. Study Parameters and Outcome

Information on the patients’ basic characteristics before surgery were collected, including age at the operation, height, weight, body mass index (BMI), alcohol consumption, cigarette use, betel nut use and past medical comorbidities. The Preoperative International Prostate Symptom Score (IPSS), prostate-specific antigen (PSA) level, prostate volume, prostate transitional zone volume and uroflowmetry results were also collected. Postoperative uroflowmetry results were collected. Preoperative uroflowmetry results within 1 year prior to the surgery and postoperative uroflowmetry results within half a year after the surgery were collected. Data on preoperative PSA level within 1 year prior to the surgery and postoperative PSA level within 1 year after the surgery were collected. The prostate volume was measured through transrectal ultrasound of the prostate (TRUS). Complication analyses include perioperative, early postoperative complication at 3 months and late postoperative complication at 12 months.

The primary outcome was the comparison of the uroflowmetry results, including the peak flow rate (Q_max_), average flow rate (Q_avg_) and post-void residual(PVR) urine between patients with prostate volume < 80 g (Group A) and prostate volume ≥ 80 g (Group B).

The secondary outcomes included the comparison of preoperative and postoperative uroflowmetry results between the two groups. The complication rates between the two groups were also analyzed.

### 2.4. Statistical Analyses

All categorical data were analyzed using the chi-squared tests and Fisher’s exact test. All data are presented as mean ± standard deviation. Efficacy analysis of uroflowmetry results was performed utilizing the Student’s *t*-test. A paired *t*-test was performed for preoperative and postoperative changes. Statistical significance was set at *p* < 0.05. Statistical analyses were performed using the Statistical Package for the Social Sciences (version 23; IBM Corp., Armonk, NY, USA).

## 3. Results

Table 1 shows the basic characteristics of the study population. The entire cohort was comprised of 232 patients. Group A included 188 patients with prostate volume < 80 g and group B included 44 patients with prostate volume ≥ 80 g. The basic characteristics of the two groups were all comparable. With the stratification of the patients into two prostate volume groups, a significant difference in the prostate volume and the prostate transitional zone volume was observed. The prostate volumes of groups A and B were 50.83 ± 14.14 g and 102.03 ± 19.36 g (*p* < 0.001), respectively. The volumes of the transitional zone of groups A and B were 24.41 ± 11.00 g and 50.81 ± 15.73 g (*p* < 0.001), respectively.

Preoperative and postoperative PSA levels are presented in Table 2. Significant differences were noted in the preoperative (6.29 ± 5.68 ng/mL vs. 12.79 ± 14.56 ng/mL, *p*: 0.012) and postoperative PSA levels (3.33 ± 3.09 ng/mL vs. 6.09 ± 3.60 ng/mL, *p* < 0.001) between groups A and B, respectively.

The uroflowmetry results are shown in Table 3 and Table 4. Variables including the Q_max_ and Q_avg_ were comparable between the two groups. The only significant difference noted between the two groups was in the postoperative PVR urine. A comparison between the uroflowmetry results pre- and postoperatively in each prostate volume group was made. Improvement was noted in all the variables including the Q_max_, Q_avg_ and PVR in both groups A and B in the uroflowmetry results.

The complication rates are presented in Table 5. None of the patients experienced perioperative complications, including transurethral resection syndrome, the need for blood transfusion or capsular perforation. After stratification of the complications into early and late complications, both groups exhibit comparable results. Moreover, analysis of the overall one-year complication rate showed no significant difference between the two groups.

## 4. Discussion

This study included 232 patients who underwent the sandwich method of bipolar TURP and GLPVP for BPH treatment. In this cohort, group A was comprised of 188 patients with prostate volume < 80 g and group B was comprised of 44 patients with prostate volume ≥ 80 g. The efficacy of the treatment in the two groups was determined using the uroflowmetry results. When postoperative improvement was analyzed between the two groups, all variables showed improvement in the uroflowmetry results. To investigate the effect of the sandwich method on a larger prostate, the pre- and postoperative variables were compared. No significant difference was observed in the peak and average flow rates pre- and postoperatively. A significantly greater PVR was noted in the postoperative group B. Considering the safety of the sandwich method, no significantly higher complication rate was noted in group B in the early, late or overall complication rates.

A higher PVR was noted in the postoperative uroflowmetry results in group B with prostate volume ≥ 80 g. PVR is an objective measurement that can be used to determine the emptying function in patients with BPH. It has been shown that a PVR > 100 mL is a predictive factor for subsequent acute urinary retention [10]. However, mixed evidence exists regarding the predictive value of PVR as a diagnostic or prognostic test. A possible reason for this may be the wide variation noted among men [11]. Thus, this might explain the significant difference in postoperative PVR observed between the two groups in this study. However, when analyzing the PVR change within the same group, both groups A and B exhibited a significant improvement in the PVR measurement.

A large prostate volume in patients who underwent TURP has been shown to be associated with an increased risk of complications [12]. Currently, for the management of the patients with large prostates, two methods have been shown to be effective according to both the EAU and AUA guidelines: HoLEP and OSP [6,8]. However, both have limitations. HoLEP is associated with a more difficult and steep learning curve and requires experience [13]. OSP, though is an effective method in patients with large prostates, is currently the most invasive surgery in patients with BPH. In a meta-analysis comparing OSP with transurethral laser prostatectomy, OSP was associated an increased hemoglobin decline, longer catheterization time, longer hospital stay and increased blood transfusion rate [14]. Thus, owing to these limitations, other treatments for large prostates should be explored.

B-TURP and GLPVP are two of the possible effective surgical treatments and are recommended for use in patients with a prostate size of 30 to 80 mL according to the EAU guidelines [8]. B-TURP has a possible shorter learning curve and allows a longer resection time than monopolar transurethral resection of the prostate [15]. GLPVP utilizes a wavelength of 532 nm, which is absorbed by the hemoglobin in prostatic tissues, to achieve a better hemostasis during the operation [15]. It is also associated with fewer surgical complications than TURP [16]. However, GLPVP, despite having a higher safety profile, is associated with a higher reoperation rate, possibly due to inadequate tissue removal [15,17].

Various studies have utilized a sandwich method with different techniques to achieve better treatment outcomes for BPH. Perk et al. first explored the sandwich method of using transurethral electrovaporization of the prostate (TUVP) and TURP in patients with BPH, and shorter catheterization time was observed in the sandwich group compared with the TURP alone group [18]. Aisuodionoe-Shadrach et al. analyzed the use of the combination of TURP with TUVP in patients with a prostate volume of 40 to 80 mL and noted significant improvements in uroflowmetry results and IPSS [19]. Zhang et al. investigated the management of patients with BPH with a prostate volume ≥ 80 g using GLPVP combined with the TUVP method and showed a significantly improved outcome [20].

In this study, a sandwich method combining B-TURP and GLPVP was utilized, and its efficacy in patients with a large prostate was assessed by comparing it with the same procedure performed in smaller prostates. The advantages of each treatment method were combined, with better hemostasis in GLPVP and a more complete tissue removal in B-TURP, and its improved efficacy in patients with larger prostates with no significant increase in complication rates was hypothesized. The results showed comparable efficacy and complication rates between the groups. Thus, the sandwich method of GLPVP and B-TURP may be a potential treatment option in patients with a larger prostate.

As with other retrospective studies, this study had some limitations. First, owing to its retrospective nature, this study is prone to selection bias. Thus, the outcome analysis was restricted, and postoperative IPSS was not incorporated into in the analysis due to limitations in the medical record review and the possible recall bias. However, this study had several strengths. The study was performed in a tertiary medical center in Taiwan, where the population was mainly comprised of Asian people. The objective outcome of uroflowmetry was analyzed in our patient groups. We have shown that the sandwich method of B-TURP and GLPVP is an effective method in patients with large prostates with no increase in complication rates.

## 5. Conclusions

In conclusion, the sandwich method of B-TURP and GLPVP may be a feasible approach for the management of patients with large prostates. However, further prospective, larger, well-designed studies are required to confirm our results.

## Figures and Tables

**Table 1:** Patient Characteristics.

Variables	Group A ** (*n* = 188)	Group B ** (*n* = 44)	*p*-Value
Age (Mean ± SD) (years)	69.06 ± 7.82	69.25 ± 6.07	0.859
Gender, Male, *n* (%)	188 (100%)	44 (100%)	
BMI, (Mean ± SD) (kg/m^2^)	24.79 ± 3.42	25.73 ± 3.23	0.101
Social History, *n* (%)			
Alcohol Consumption	25 (13.3%)	5 (11.4%)	0.669
Betel Nut Use	12 (6.4%)	6 (13.6%)	0.123
Cigarette Use	43 (22.9%)	13 (29.5%)	0.411
Preoperative IPSS	23.55 ± 4.40	23.83 ± 4.50	0.754
Comorbidities, *n* (%)			
Hypertension	76 (40.4%)	24 (54.5%)	0.089
Diabetes Mellitus	32 (17.0%)	7 (15.9%)	0.859
Gastrointestinal Disorders	18 (9.6%)	2 (4.5%)	0.381
Dyslipidemia	12 (6.4%)	4 (9.1%)	0.513
Coronary Artery Disease	13 (6.9%)	1 (2.3%)	0.479
Prostate Volume (g)	50.83 ± 14.14	102.03 ± 19.36	<0.001 *
Prostate Transitional Zone Size (g)	24.41 ± 11.00	50.81 ± 15.73	<0.001 *

* *p*-value < 0.05; ** Group A: Prostate volume < 80 g; Group B: Prostate volume ≥ 80 g; BMI: Body mass index; SD: Standard deviation; IPSS: International prostate symptom score.

**Table 2:** Biochemical Values.

Variables	Group A **	Group B **	*p*-Value
Preoperative			
Total PSA (ng/mL)	6.29 ± 5.68	12.79 ± 14.56	0.012 *
Postoperative			
Total PSA (ng/mL)	3.33 ± 3.09	6.09 ± 3.60	<0.001 *

* *p*-value < 0.05; ** Group A: Prostate volume < 80 g; Group B: Prostate volume ≥ 80 g; PSA: Prostate specific antigen.

**Table 3:** Uroflowmetry between different prostate volume.

Variables	Group A **	Group B **	*p*-Value
Preoperative			
Q_max_ (mL/s)	9.26 ± 7.22	8.07 ± 3.01	0.379
Q_avg_ (mL/s)	3.46 ± 1.87	3.07 ± 1.17	0.152
PVR	125.88 ± 166.99	102.77 ± 76.76	0.258
Postoperative			
Q_max_ (mL/s)	16.05 ± 8.48	14.32 ± 7.52	0.250
Q_avg_ (mL/s)	7.37 ± 4.20	6.05 ± 3.61	0.077
PVR	32.30 ± 45.02	53.33 ± 39.23	0.008 *

* *p*-value < 0.05; ** Group A: Prostate volume < 80 g; Group B: Prostate volume ≥ 80 g; Q_max_: Peak flow rate; Q_avg_: Average flow rate; PVR: Postvoid residual.

**Table 4:** Uroflowmetry in different prostate volume.

Variables	Preoperative	Postoperative	*p*-Value
Group A **			
Q_max_ (mL/s)	9.53 ± 7.63	16.07 ± 8.39	<0.001 *
Q_avg_ (mL/s)	3.53 ± 1.88	7.39 ± 4.27	<0.001 *
PVR	131.89 ± 176.28	25.60 ± 32.64	0.001 *
Group B **			
Q_max_ (mL/s)	7.92 ± 3.16	14.12 ± 8.00	<0.001 *
Q_avg_ (mL/s)	3.00 ± 1.23	5.92 ± 3.77	<0.001 *
PVR	104.35 ± 81.66	49.08 ± 34.52	0.001 *

* *p*-value < 0.05; ** Group A: Prostate volume < 80 g; Group B: Prostate volume ≥ 80 g; Q_max_: Peak flow rate; Q_avg_: Average flow rate; PVR: Postvoid residual.

**Table 5:** Complications.

Variables	Group A *	Group B *	*p*-Value
Perioperative			
TUR Syndrome	0	0	
Blood Transfusion	0	0	
Capsular Perforation	0	0	
ER Visit-1 year	15	4	0.765
Readmission-1 year	9	2	1.000
Reintervention-1 year	9	2	1.000
BPH Recurrence	0	1	0.190
Urethral Stricture	3	1	0.571
Bladder Neck Stenosis	5	0	0.586
Hematuria	1	0	1.000
Incontinence	0	0	
Early Complications **			
ER Visit	13	2	0.742
Readmission	5	2	0.620
Reintervention	3	2	0.241
Late Complications **			
ER Visit	2	2	0.165
Readmission	4	0	1.000
Reintervention	6	0	0.598

* Group A: Prostate volume < 80 g; Group B: Prostate volume ≥ 80 g; ** Early complications: 0–3 months; Late complications: 4–12 months.

## Data Availability

The data presented in this study are available on request from the corresponding author.
